# Poxvirus Infections in Common Swifts (*Apus apus*)

**DOI:** 10.3390/vetsci13080768

**Published:** 2026-07-31

**Authors:** Marko Legler, Kristin Heenemann, Ingo Gerhauser

**Affiliations:** 1 Department of Small Mammal, Reptile and Avian Medicine and Surgery, University of Veterinary Medicine Hannover, Foundation, Bünteweg 9, 30559 Hanover, Germany; 2 Institute of Virology, Centre for Infectious Diseases, Faculty of Veterinary Medicine, Leipzig University, An den Tierkliniken 29, D-04103 Leipzig, Germany; kristin.heenemann@vetmed.uni-leipzig.de; 3 Department of Pathology, University of Veterinary Medicine Hannover, Foundation, Bünteweg 17, 30559 Hanover, Germany; ingo.gerhauser@tiho-hannover.de

**Keywords:** swift, virus infection, *Apodidae*, *Avipoxvirus*

## Abstract

Members of the Genus *Avipoxvirus* have been reported in more than 374 avian species worldwide. Generally, poxviruses are considered to have a limited host range; however, canarypox-like viruses appear to infect a broader host range. In 2024, four juvenile common swifts in an age of 30 to 35 days from three different breeding sites of Lower Saxony, Germany, showed nodular, verrucous and crusted skin lesions of the wings, especially of the dorsal surface of the elbow and forearm region and the skin between the outer primary flight feathers. The skin on the head or the feet as well as the mucous membrane of the oral cavity was not affected. Two swifts of these cases had to be euthanized due to pox-related alterations of feather follicles of the primary flight feathers and associated feather growth disorders and loos. The other two swifts were released into the wild after recovery and successful hand-rearing. The diagnosis of a poxvirus infection is based in our cases on a combination of clinical examination, histology, transmission electron microscopy, and molecular biological investigations. The histological examination of skin lesions displayed severe heterophilic and histiocytic dermatitis with severe epithelial hyperplasia, mild orthokeratotic hyperkeratosis and cytoplasmic eosinophilic inclusion bodies (Bollinger’s inclusion bodies) within frequently swollen keratinocytes (ballooning degeneration). Using transmission electron microscopy, virions showing typical brick-shaped poxvirus morphology were visualized. *Avipoxvirus*-specific DNA was detected by PCR. The sequence analysis of the 176 bp amplicon revealed 100% identity to a canarypox virus and a shearwaterpox virus 2 strain. Herpesvirus- or papillomavirus-specific DNA was not detected by PCR. To our knowledge, this is the first report of an avipoxvirus infection in a swift species. Notably, in all swift cases, the disease was limited to the skin of the wings. The origin of the virus and its transmission to swifts are unknown. However, transmission from other wild birds via biting insects appears to be possible, as has been described for canarypox-like viruses.

## Simple Summary

The knowledge about viral infections in swifts is limited. During the breeding season, swifts leave their exclusively aerial lifestyle. As a result, they become more vulnerable to infectious diseases, especially those transmitted by vectors. In a wildlife rescue center specialized for swifts, four age-related flightless juvenile common swift (*Apus apus*) of three different breeding sites were admitted with nodular, verrucous and crusted skin lesions of the wings. Histopathology and molecular diagnostic investigations, including polymerase chain reaction (PCR), confirmed an avipoxvirus infection. Feather follicle damage of primary flight feathers due to the poxvirus infection and the resulting impaired feather growth, which precluded a release, led to the euthanasia of two birds. The other two swifts were successfully released into the wild following recovery from the skin lesions and successful hand-rearing.

## 1. Introduction

Avian pox is caused by members of the genus *Avipoxvirus* (APV) and is a widespread infectious disease affecting poultry, pet and wild birds [1,2,3]. Infections can result in a diphtheritic (wet pox) or cutaneous (dry pox) disease form, which is likely dependent on the poxvirus and the diseased bird species [3]. The diphtheritic form is characterized by proliferative and fibrino-necrotic alterations of the mucous membranes of the oral cavity and the respiratory tract [4,5]. The more common cutaneous form of an APV infection shows nodular and wart-like lesions on the non-feathered skin. Commonly, poxviruses are transmitted by biting arthropods (vector-borne mechanical transmission) or through direct contact with infected birds [3,6,7,8]. Spread via contaminated objects (fomites) or aerosol has also been described [3,9,10]. Aerosol transmission is associated with the diphtheritic form affecting the respiratory tract [9]. Infected wild birds are considered the main reservoir of APV [3].

The genus *Avipoxvirus* belongs to the subfamily *Chordopoxvirinae* within the family *Poxviridae*. Poxviruses are large, linear, double-stranded DNA viruses. Their genome consists of 250–400 kb and encodes 250–350 proteins [3]. Genetic analyses of APV often use the 578 bp 4b gene fragment which is relatively conserved between different strains [11,12]. Genetically, APV can be classified into different major clades: fowlpox-like (A), canarypox-like (B) and psittacinepox-like (C) viruses [1,3,11]. APV infections have been detected in more than 374 bird species from 23 orders [1]. Adaptation of APV strains to specific host taxa is thought to be restricted to only in a subset of strains [11,13,14,15]. In particular, canarypox-like viruses are believed to exhibit a broader host range, possibly related to their genetic composition [16,17].

The pathogenicity of APV may differ depending on the APV strain, host species, and individual immune status of the host. In general, cases presenting with the diphtheritic form show higher mortality rates compared to those with an exclusively cutaneous manifestation [4,11,18].

To the best of the authors’ knowledge, there is no information about APV infection in swift species (*Apodidae*) worldwide [1,2,3,11,13]. The common swift (*Apus apus*) is the most frequently recorded representative of the order *Apodiformes* in Germany. As a long-distance migrant, the common swift overwinters in sub-Saharan Africa from September to May. During this period, common swifts remain almost continuously airborne for up to ten months per year, displaying highly specialized aerial adaptations and behavior [19,20,21,22,23,24,25,26,27]. In Germany, with 215,000 to 395,000 breeding pairs, the common swift is classified as of least concern and a frequent patient at wildlife rehabilitation centers [26,27,28,29]. During their relatively short three-month breeding season, common swifts are exposed to potential disease vectors, including biting and blood-feeding insects. Consequently, they may acquire infections that are less likely during the rest of the year, when their predominantly aerial lifestyle limits such exposure. In the context of wildlife rehabilitation, current knowledge of infectious diseases in common swifts and the potential transmission of pathogens from wildlife populations to common swifts remains limited [28,30,31,32].

This communication represents the first cases of APV infections in common swifts (*Apus apus*) of different breeding locations in Germany.

## 2. Case Report

### 2.1. Clinical Cases

Four juvenile common swifts (S1 to S4) presenting with varying degrees of cutaneous wing lesions were admitted to a wildlife rescue center specialized for swifts in Lower Saxony, Germany, during a heatwave in August 2024. The swifts originated from three different breeding sites in Lower Saxony (Table 1), with S1 and S2 originating from the same breeding colony. Based on physical and feather development, all swifts were estimated to be between 30 and 35 days old and were in good body condition, weighing 36 to 42 g. However, the juvenile swift S1 showed a poor general condition at the time of administration. 

**Figure 1:**
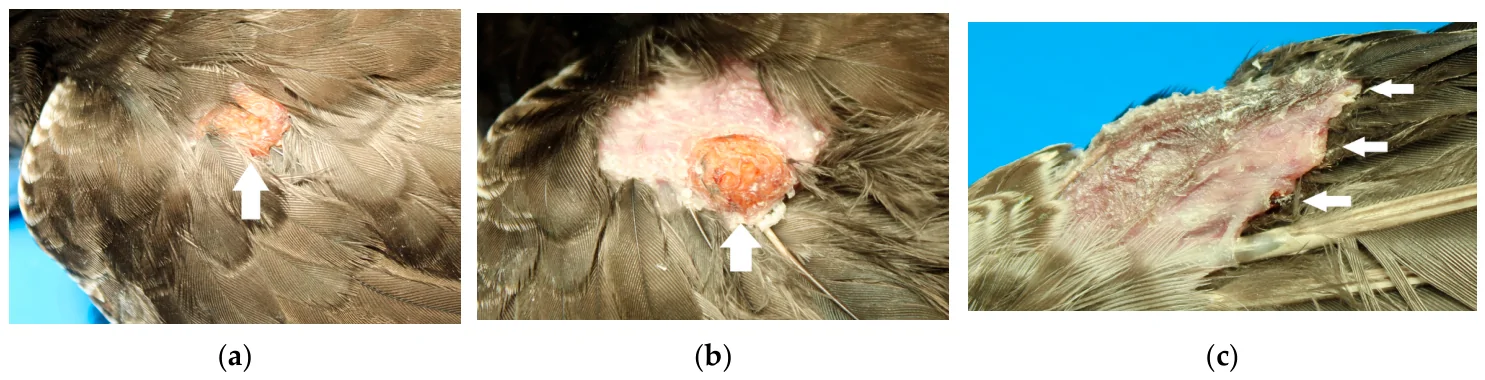

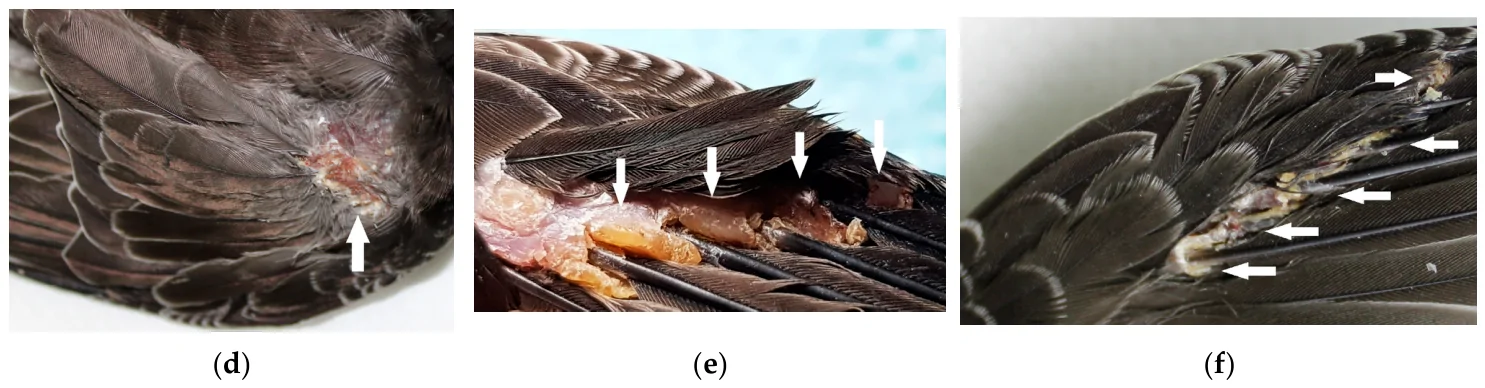
Avipoxvirus infection in juvenile common swifts (S1 and S2), with nodular, verrucous and crusted skin lesions (arrows): (**a**,**b**) in the elbow region before and after plucking of the covert feathers to expose the pox lesion, swift S1; (**c**) lesions in the region of the feather follicles of the primary flight feathers of the left wing with pox-related damage of the follicles and feather loss (covert feathers removed), swift S1; (**d**) lesions in the forearm region in the process of healing, swift S2; (**e**) lesions in the region of the primary flight feathers of the right wing at the time of admission; and (**f**) the same region after a week, showing pox-related damage of the feather follicles during the process of healing, swift S2.

In all birds, the skin lesions were characterized by nodular, verrucous and crusted skin lesions of varying severity and ulceration (Figure 1). Between 2023 and 2025, comparable findings were identified in only the four swifts presented here among 920 examined common swifts from an age of 10 days to adult (2023: 228 swifts; 2024: 336 swifts; 2025: 356 swifts). The lesions affected only the skin of the wings, the dorsal surface of the elbow and forearm region and the areas between the primary flight feathers (S1: left wing: single lesion in elbow region 4 × 5 mm and skin between P7 to P10; S2: both wings: single lesion middle forearm region 5 × 5 mm and skin between P5 to P10; Figure 1). The skin of the head and the feet as well as the mucous membrane of the oral cavity were not affected.

The cutaneous alterations between the primary feathers affected the feather follicles and caused impaired feather growth in swifts S1 and S2, with partial loss of the flight feathers (Figure 1). The birds S1 and S2 were euthanized due to their already heavily destroyed feather follicles, and without any prospect of normal feather regrowth and successful rehabilitation. These two birds were further examined in a pathological examination with full necropsy and histopathology (S1 and S2), transmission electron microscopy (S1) and virological examinations (S1 and S2; Table 1).

**Figure 2:**
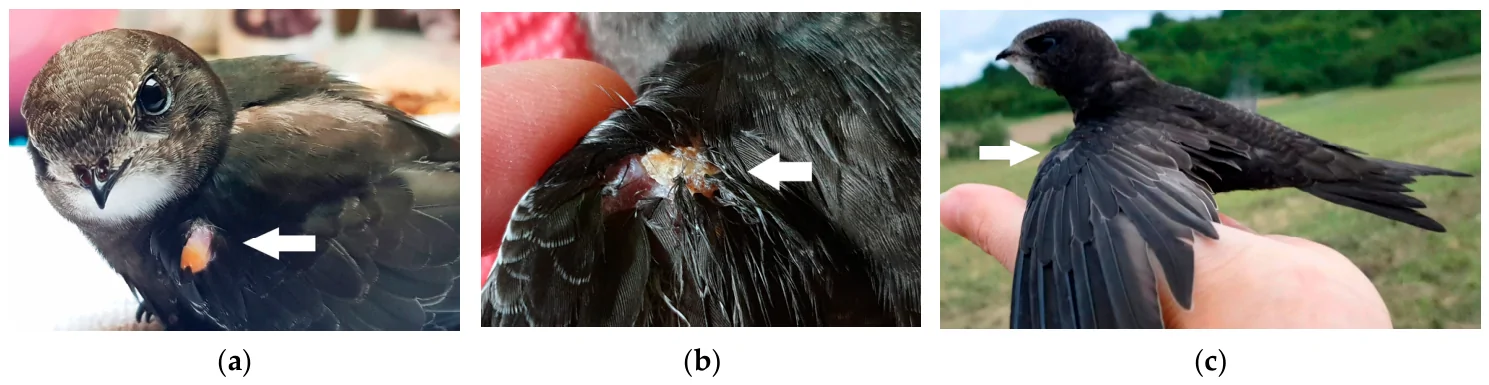
Suspected avipoxvirus infection in juvenile common swifts, S3 and S4. Verrucous and crusted skin lesions of the left wings (arrow): (**a**) at the time of admission (S4), (**b**) during the healing process (S3) and (**c**) after recovery, at the time of release; a small featherless area is still visible (S3).

Swift S3 showed a single nodular verrucous lesion (4 × 4 mm) on the cranial dorsal surface of the forearm of the left wing and swift S4 a single crusted skin lesion (4 × 3 mm) on the middle surface of the left forearm (Figure 2). A poxvirus infection was suspected based on the clinical presentation in these cases. The cutaneous lesions of both of these swifts resolved without additional treatment within three weeks (Figure 2). The skin healed and regenerated successfully, although no signs of feather regrowth were observed prior to the swifts release. However, following successful hand-rearing, both swifts were released back into the wild. During hand-rearing, the birds were housed together at room temperature and fed with a diet based on steppe crickets (90%), wax moth larvae and drone brood (10%) supplemented with Korvimin ZVT (WDT, Garbsen, Germany) according to Haupt (2001) [33]. Vitamin B complex (30.0 mg/kg; Serumwerk Bernburg AG, Bernburg, Germany) was subcutaneously supplemented every 10 days to prevent hypovitaminosis B as described for swifts [28,29,33]. The detached crusts of S3 and S4 after healing were collected by the rehabilitator for a virological examination.

### 2.2. Pathological Investigation

**Figure 3:**
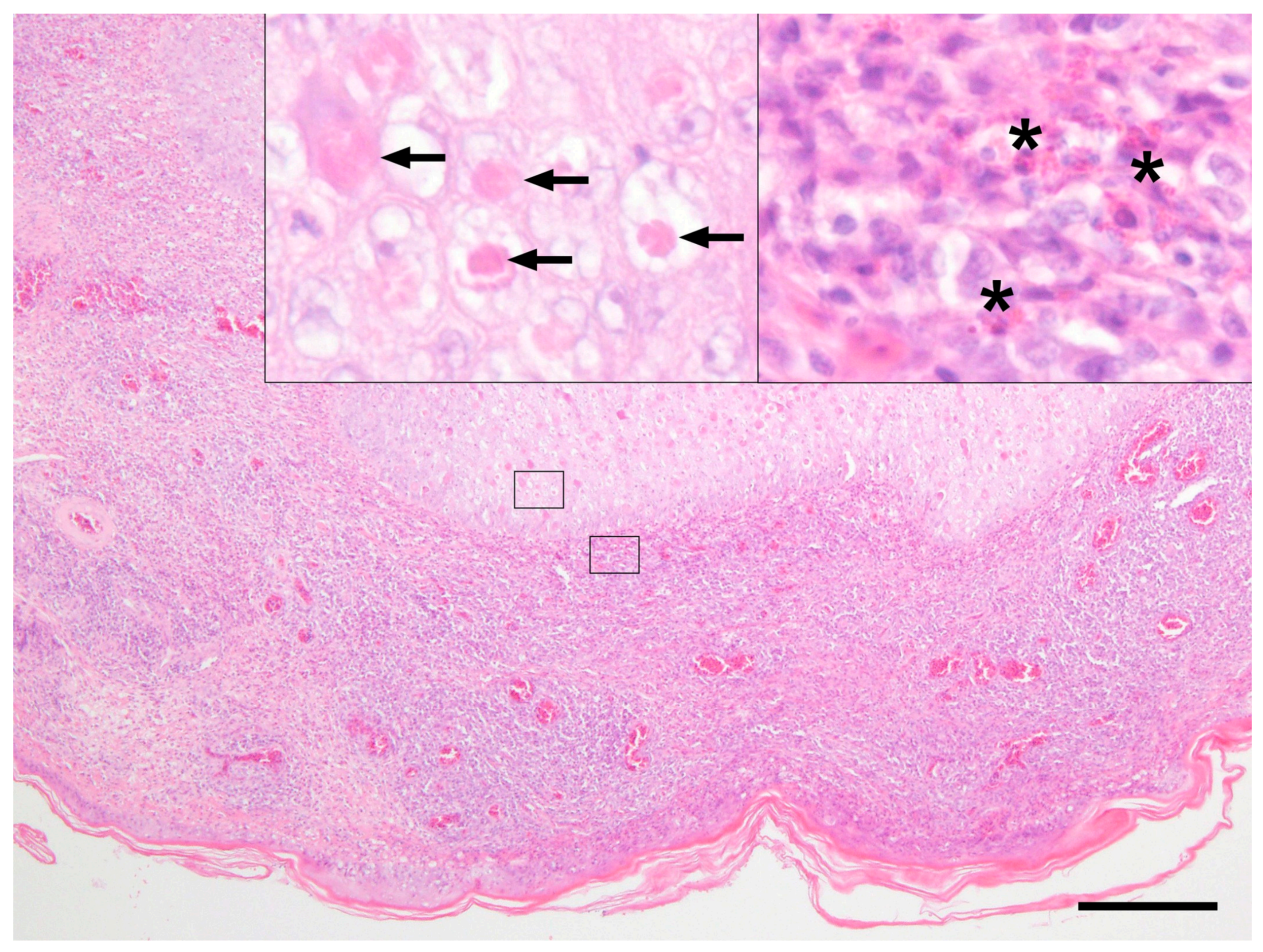
Avipoxvirus infection in a juvenile common swift (S1). Histology of a skin lesion with severe heterophilic and histiocytic dermatitis, severe epithelial hyperplasia, and mild orthokeratotic hyperkeratosis. (**Left inset**): Large cytoplasmic eosinophilic inclusion bodies (Bollinger’s inclusion bodies, arrows) and swollen keratinocytes. (**Right inset**): Dermal inflammatory infiltrates are dominated by heterophils (asterisks) and macrophages. Bar = 200 µm in the overview and 20 µm in the insets. HE staining.

The macroscopic examination of the organs of the swift S1 and S2 revealed no abnormalities in a full necropsy. There were no indications of a diphtheritic disease form of the mucous membranes of the oral cavity or respiratory tract in both necropsied birds. Skin lesions and tissue samples of the heart, lung, liver, kidney, spleen, small intestine with pancreas, mucosa of oral cavity and cloaca were fixed in 10% formalin, routinely processed and embedded in paraffin wax. Tissue sections of 3 µm thickness were stained with hematoxylin and eosin (HE). The main findings were observed on the skin. The histological examination of the skin lesions displayed severe heterophilic and histiocytic dermatitis with severe epithelial hyperplasia, mild orthokeratotic hyperkeratosis and 5–10 µm cytoplasmic eosinophilic inclusion bodies (Bollinger’s inclusion bodies) within frequently swollen keratinocytes (ballooning degeneration). Few lymphocytes and plasma cells were also present in the dermal inflammatory infiltrates (S1; Figure 3).

**Figure 4:**
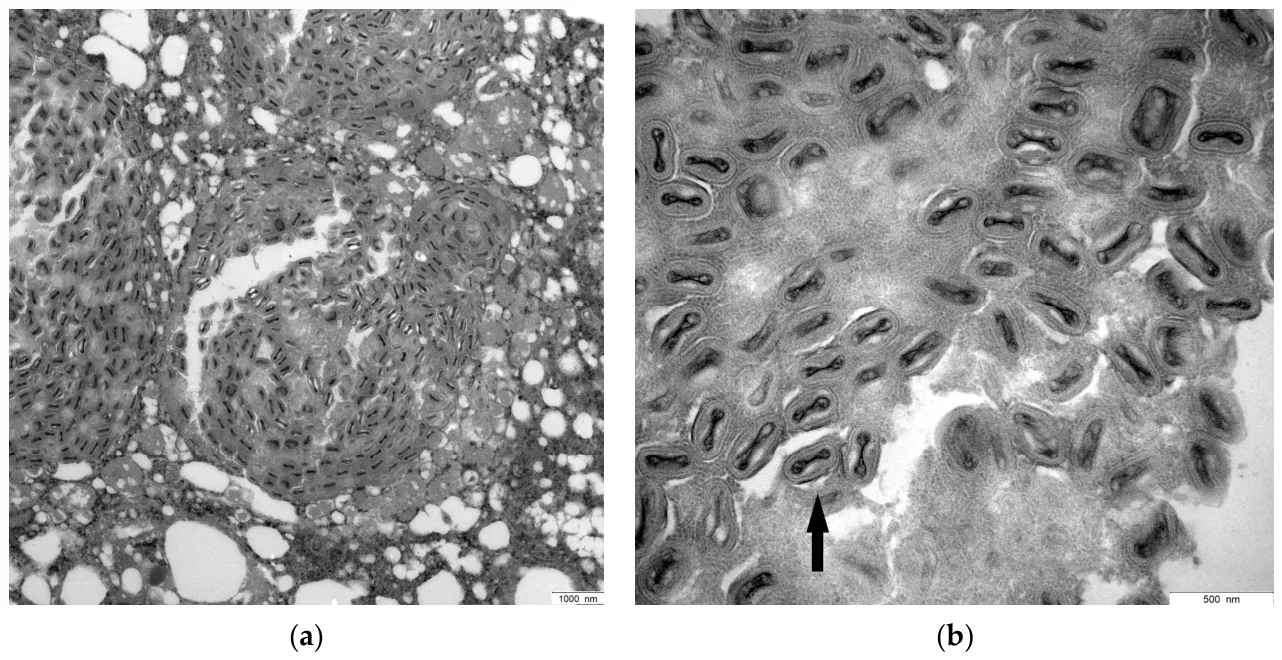
Transmission electron microscopy of the skin lesion of a juvenile common swift (S1, Figure 1a,b) with an avipoxvirus infection (S1). Keratinocyte with numerous brick-shaped virus particles occasionally displaying a biconcave (dumbbell-shaped) structure (arrows); pop-off technique; bar 1000 nm (**a**) and bar 500 nm (**b**).

Transmission electron microscopy of the skin lesion of S1 using the pop-off technique [34] revealed multiple biconcave brick-shaped enveloped virus particles showing typical poxvirus morphology measuring approximately 230–330 nm (Figure 4). The virus particles contained granular material within a dumbbell-shaped central body and two lateral bodies.

### 2.3. Virological Investigation

For further virological examination, the skin lesions and organ samples (lung, liver, spleen and kidney) of each swift (S1 and S2) were pooled and stored at −80 °C until further molecular diagnostic investigation. From swifts S3 and S4, only loose scabs collected after healing were available and were pooled for examination. DNA was extracted from the tissue and scab samples using the DNeasy Blood & Tissue Kit (Qiagen, Hilden, Germany) according to the manufacturer’s instructions. DNA concentration was determined using a NanoDrop 2000c spectrophotometer (Thermo Fisher Scientific, Wilmington, DE, USA). Samples were screened for avipoxvirus, papillomavirus, and herpesvirus DNA by PCR. Avipoxvirus DNA was initially investigated using the PCR assay described by Lüschow et al. [12]. In addition, a second PCR assay for avipoxvirus DNA detection was performed according to Pérez-Tris et al. [35]. Papillomavirus DNA was investigated according to Husnjak et al. [36], and herpesvirus DNA was analyzed using the consensus primer PCR described by van Devanter et al. [37]. The PCR assay according to Pérez-Tris et al. [35] employed the primer pair FORM29-1 (5′-TGTAAAAGCCAGTCATCCTC-3′) and FORM29-2 (5′-CACCGAATAAAGCTACCAATA-3′), targeting a fragment of the avipoxvirus 4b gene. Positive PCR products were purified using the GenJet PCR Purification Kit (Thermo Fisher Scientific, Waltham, MA, USA) and subsequently sequenced using the dideoxy chain-termination method according to Sanger. Nucleotide sequences were analyzed and edited using the GENtle program (Magnus Manske, Cologne, University of Cologne, Cologne, Germany). Sequence similarity searches were performed using the Basic Local Alignment Search Tool (BLAST+ 2.16.0) of the National Center for Biotechnology Information (NCBI; https://www.ncbi.nlm.nih.gov/, accessed on 7 April 2025).

Avipoxvirus DNA was detected in the skin lesion of swift S2 using the PCR assay according to Pérez-Tris et al. [34], yielding an amplicon of 179 base pairs (bp). The sequence was deposited in the NCBI GenBank under NCBI accession no.: PZ469463. The NCBI Blast analysis revealed a 100% nucleotide sequence identity of the obtained sequence (179 bp) to a Canarypox virus (NCBI accession no.: NC_005309.1) and a Shearwaterpox virus 2 strain (NCBI-accession no.: KX 857215.1). No avipoxvirus DNA was detected in the pooled samples of swift S1 or in the scabs samples collected after healing from the swifts S3 and S4. Herpes- and papillomavirus-specific DNA were not detected by PCR in the examined samples of swifts S1 and S2.

## 3. Discussion

The cases described here represent the first documented avipoxvirus infection in swifts. The diseased juvenile common swifts were presented with the cutaneous form of avipox, characterized by nodular, verrucous, and crusted skin lesions, affecting the dorsal surface of the elbow and forearm region and the areas between the primary flight feathers of the wings. In contrast to many other avian species, the featherless areas of the legs and the base of the beak, which are typically affected by pox lesions, remained unaffected in the examined swifts [1,3,32]. No evidence of the diphtheritic form was observed. This finding differs from reports in canaries, in which diphtheritic lesions are frequently associated with canarypox virus infections [5,11]. Thus, the clinical manifestation observed in common swifts shows some similarities to that reported in hummingbirds (*Trochilidae*, *Apodiformes*), in which skin lesions, including those affecting the wings, occur predominantly [38,39].

The reasons for the unusual distribution of pox lesions in common swifts remain unclear. One possible explanation may be related to species-specific anatomical characteristics during juvenile development. In growing swifts, the developing wings represent a disproportionately large body surface area and may therefore be particularly exposed to biting arthropods, which are considered the main vectors of avipoxviruses [3,6,7,8,28]. Similar observations have been reported in great tits, where pox lesions frequently occurred in some regions of the body [13,40].

In our cases, the diagnosis of pox in swifts was particularly reliable, based on histopathological examination, in which inclusion bodies (Bollinger’s inclusion bodies) could be identified, in combination with molecular biological investigations (PCR). Using TEM, the typical structure of the poxviruses could be visualized, thereby confirming the histological diagnosis (Figure 4) [34]. The complementary use of histopathology, TEM, and PCR strengthened the diagnostic confirmation of APV infection where appropriate samples were available.

PCR can be used to diagnose a poxvirus infection and to further characterize the origin of the infection. Avipoxvirus DNA was detected by PCR only in swift S2. The main challenges arise from obtaining suitable sample material. In swifts, the affected skin areas are relatively small, and samples with a very low viral load may therefore lead to false-negative PCR results. Furthermore, the swifts were already in the recovery phase, which may have reduced the viral load in the collected samples. In addition, samples from swifts S3 and S4 consisted only of crusts that had detached after healing. These samples are also assumed to contain a low viral load. These factors may explain the negative PCR results obtained in some clinically affected birds. Studies in hummingbirds have confirmed PCR of skin swabs as a suitable screening method for poxvirus infections in these species [39]. Detection by PCR was achieved primarily using the previously established primers by Perez-Tris et al. [35], which have also been applied for the detection of poxviruses in challenging sample materials such as museum skins. Ultimately, this method also led to the detection of a poxvirus in our cases.

APV can be additionally isolated from tissue samples by inoculation into the chorioallantoic membrane of specific pathogen-free chicken embryos or can be cultured using, for example, chicken embryo fibroblasts [1,32]. These methods were not pursued further in our investigations due to the limited quantity and poor quality of the sample material.

The APV detected in a swift in our cases showed 100% nucleotide identity over the analyzed P4b gene fragment to canarypox virus and shearwaterpox virus 2 sequences, with both viruses belonging to the canarypox-like avipoxvirus lineage [5,17,41]. However, due to the short length of the obtained sequence fragment in our cases, a robust phylogenetic classification of the detected virus was not possible.

However, canarypox-like avipoxviruses are known, partly due to their genetic constitution, to overcome species barriers and to possess a broad host range [11,16,41,42,43]. This is also reflected in other studies; for example, the shearwater poxvirus was detected in a Chilean flamingo in Hanover [41] and invasive and pathogenic APV infections have been found in naïve bird populations on island groups [17,43]. This indicates that these viruses circulate in the wild bird populations and can infect species that are less commonly affected by a poxvirus infection [11]. It is therefore possible that this was also the case in our APV infections in common swifts and the detected virus is not a swift-specific poxvirus. The available literature indicates that mechanical transmission of APV via biting insects appears to play an important role in the distribution [6,7]. It is reported that the prevalence of APV infections increases after vector population peaks [44]. In particular, the population density of mosquitos was estimated to be very high in 2024, the year in which our cases were observed. In that year, numerous West Nile and Usutu virus infections were also observed in wild and captive birds in Germany, which are also transmitted by mosquitoes [44]. Another possible route of transmission of APV in swifts could be via the swift louse fly (*Crataerina pallida*). To date, there is no evidence in the literature for the presence of poxviruses in the swift louse fly. However, poxviruses have been detected in louse flies collected from other bird species [45]. To date, no adult swifts with poxvirus infections have been recorded. Subclinical infections may also occur in swifts but, in contrast to other bird species, have not yet been described [2]. Age and immune function are important for the development of the poxvirus disease. The majority of studies suggest that avipox lesions are more frequent in juvenile birds [46,47].

Clinical experience indicates that pox infections confined exclusively to the skin have a better prognosis for complete recovery [1,32,39]. In the cases presented here, two animals exhibiting clinical signs of a pox infection recovered and were successfully released back into the wild. Complications due to bacterial or fungal infections reported in the literature were not observed in the swift cases examined in our study [1]. Unfortunately, in two animals, the feather follicles were so severely affected that normal regrowth of the primary flight feathers could not be expected, and the birds had to be euthanized. Poxvirus infections can result in functional impairment of the affected and adjacent tissues due to inflammation-induced tissue damage, ulceration and subsequent scar formation. In our cases, this is manifested by the altered feather follicles and feather loss. In other bird species, particularly raptors, this process may, for example, lead to the loss of distal phalanges or claws [32]. Important differential diagnoses, such as a fungal infection or a neoplastic lesion, could be excluded histologically in two of our cases [32].

The prevalence of APV infections among wild birds, and especially in swifts, is completely unknown [1,2,3,32]. As these are the first reported APV cases in swifts over decades of research on this bird species, it can be assumed that these clinically apparent infections represent rather sporadic events. According to our data, the prevalence of clinically apparent poxvirus disease ranges in common swifts from 0 to 1.2%, which is low compared with that reported for other bird species, for example as in tits [1,3,11,13]. However, these data do not necessarily reflect the true prevalence of poxvirus infection; rather, they only provide an indication of its occurrence in swifts presenting with trauma and diseases during the breeding season ranging in age from 10 days old to adults. However, climatic changes can lead to an increase in the relevant vectors, and/or to a rise in poxvirus infections in the wild bird population. As a result, poxvirus infections in swifts may become more common in the future [40,43]. The occurrence of poxvirus infections at widely separated breeding sites indicates that the common swift is a susceptible species for an APV infection and that the changing environmental conditions may facilitate such events.

In conclusion, these are the first cases of poxvirus infection of common swifts in connection with nodular, verrucous and crusted skin lesions of the wings. The detected avipoxvirus showed the highest sequence similarity to canarypox virus and shearwaterpox virus 2 over the analyzed P4b gene fragment. Therefore, transmission from other wild birds to swifts through biting insects appears likely. Further studies are necessary to understand the distribution, transmission, and significance of viral infections in swifts.

## Figures and Tables

**Table 1:** Common swift cases included in the study.

Swift	Date Admitted	Location (City)	Age (Days)	Lesions Located	Diagnostic
S1	09.08.24	Neustadt am Rübenberge	30–35	left wing	Clin. exam.; Histopathology; TEM; PCR
S2	14.08.24	Hannover	30–35	right/left wing	Clin. exam.; Histopathology; PCR
S3	11.08.24	Neustadt am Rübenberge	30–35	left wing	Clin. exam.; PCR
S4	16.08.24	Göttingen	30–35	left wing	Clin. exam.; PCR

PCR—polymerase chain reaction; TEM—transmission electron microscopy; Clin. exam.—clinical examination.

## Data Availability

The original contributions presented in this study are included in the article. Further inquiries can be directed to the corresponding author.

## References

[1] Williams R.A.J., Truchado D.A., Benitez L. (2021). A Review on the Prevalence of Poxvirus Disease in Free-Living, Captive and Wild Birds. Microbiol. Res..

[2] Bertelloni F., Ceccherelli R., Marzoni M., Poli A., Ebani V.V. (2022). Molecular Detection of *Avipoxvirus* in Wild Birds in Central Italy. Animals.

[3] van Riper C., Forrester D., Thomas N.J., Hunter D.B. (2007). Avian Pox. Infectious Diseases of Wild Birds.

[4] Shearn-Bochsler V., Green D.E., Converse K.A., Docherty D.E., Thiel T., Geisz H.N., Fraser W.R., Patterson-Fraser D.L. (2008). Cutaneous and diphtheritic avian poxvirus infection in a nestling Southern Giant Petrel (*Macronectes giganteus*) from Antarctica. Polar Biol..

[5] Reza K., Nasrin A., Mahmoud S. (2013). Clinical and pathological findings of concurrent poxvirus lesions and aspergillosis infection in canaries. Asian Pac. J. Trop. Biomed..

[6] Yeo G., Wang Y., Chong S.M., Humaidi M., Lim X.F., Mailepessov D., Chan S., How C.B., Lin Y.N., Huangfu T. (2019). Characterization of Fowlpox virus in chickens and bird-biting mosquitoes: A molecular approach to investigating avipoxvirus transmission. J. Gen. Virol..

[7] Kligler I.J., Aschner M. (1931). Demonstration of presence of fowl pox virus in wild caught mosquitoes (*Culex pipiens*). Proc. Soc. Exp. Biol. Med..

[8] Huong C.T.T., Murano T., Uno Y., Usui T., Yamaguchi T. (2014). Avian pathology molecular detection of avian pathogens in poultry red mite (*Dermanyssus gallinae*) collected in chicken farms. J. Vet. Med. Sci..

[9] Mete A., Borst G.H.A., Dorrestein G.M. (2001). Atypical poxvirus lesions in two Galapagos doves (*Nesopelia g. galapagoensis*). Avian Pathol..

[10] Wilcoxen T.E., Horn D.J., Hogan B.M., Hubble C.N., Huber S.J., Flamm J., Knott M., Lundstrom L., Salik F., Wassenhove S.J. (2015). Effects of bird-feeding activities on the health of wild birds. Conserv. Physiol..

[11] Bolte A.L., Meurer J., Kaleta E.F. (1999). Avian host spectrum of avipoxviruses. Avian Pathol..

[12] Lüschow D., Hoffmann T., Hafez H.M. (2004). Differentiation of avian poxvirus strains on the basis of nucleotide sequences of 4b gene fragment. Avian Dis..

[13] Bosch S. (2017). Vogelpocken bei Kohlmeisen *Parus major* und andere Vogelarten in Südwestdeutschland 2002–2016. Ornithol. Mitteilungen.

[14] Gyuranecz M., Foster J.T., Dan A., Ip H.S., Egstad K.F., Parker P.G., Higashiguchi J.M., Skinner M.A., Hofle U., Kreizinger Z. (2013). Worldwide phylogenetic relationship of avian poxviruses. J. Virol..

[15] Jarmin S., Manvell R., Gough R.E., Laidlaw S.M., Skinner M.A. (2006). Avipoxvirus phylogenetics: Identification of a PCR lengthpolymorphism that discriminates between the two major clades. J. Gen. Virol..

[16] Oliveira G.P., Rodrigues R.A.L., Lima M.T., Drumond B.P., Abrahão J.S. (2017). Poxvirus Host Range Genes and Virus-Host Spectrum: A Critical Review. Viruses.

[17] Thiel T., Whiteman N.K., Tirapé A., Baquero M.I., Cedeño V., Walsh T., Uzcátegui G.J., Parker P.G. (2005). Characterization of canarypox-like viruses infecting endemic birds in the Galápagos Islands. J. Wildl. Dis..

[18] Donnelly T.M., Crane L.A. (1984). An epornitic of avian pox in a research aviary. Avian Dis..

[19] Åkesson S., Klaassen R.R., Holmgren J., Fox J.W., Hedenström A. (2012). Migration routes and strategies in a highly aerial migrant, the common swift *Apus apus*, revealed by light-level Geolocators. PLoS ONE.

[20] Hedenström A., Norevik G., Warfvinge K., Andersson A., Bäckman J., Åkesson S. (2016). Annual 10-Month Aerial Life Phase in the common swift *Apus apus*. Curr. Biol..

[21] Holmgren J. (2004). Roosting in tree foliage by common swift *Apus apus*. Ibis.

[22] Muijres F.T., Henningsson P., Stuiver M., Hendenström A. (2012). Aerodynamic flight performance in flap-gliding birds and bats. J. Theor. Biol..

[23] Rattenborg N.C., Voirin B., Cruz S.M., Tisdale R., Dell’Omo G., Lipp H.-P., Wikelski M., Vyssotski A.L. (2016). Evidence that birds sleep in mid-flight. Nat. Commun..

[24] Wellbrock A.H.J., Bauch C., Rozman J., Witte K. (2017). “Same procedure as last year?”—Repeatedly tracked swifts show individual consistency in migration pattern in successive years. J. Avian Biol..

[25] Glutz von Blotzheim U.N., Bauer K.M. (1997). Handbuch der Vögel Mitteleuropas (Band 9).

[26] Gedeon K., Grüneberg C., Mitschke A., Sudfeldt C., Eickhorst W., Fischer S., Flade M., Frick S., Geiersberger I., Koop B. (2014). Atlas Deutscher Brutvogelarten, Atlas of German Breeding Birds.

[27] Tigges U. (2006). The breeding cycle in calendar form of the common swift *Apus apus* across its Eurasian breeding range—A testable hypothesis?. Podoces.

[28] Haupt C. (2009). Radiologische Diagnostik am Mauersegler (*Apus apus* Linnaeus, 1758): Anatomie und Pathologie des Skeletts und ein Beitrag zur Tierärztlichen Therapie und Prognose. Doctoral Dissertation.

[29] Matthes H. (2006). Recovery of a hand-reared common swift (*Apus apus*). APUSlife.

[30] Tiyawattanaroj W., Jung A., Mohr L., Legler M. (2021). Examination of common swifts (*Apus apus*) for salmonella shedding in the area of Hannover, Lower Saxony, Germany. Tierarztl. Prax. Ausg. K Kleintiere Heimtiere.

[31] Tiyawattanaroj W., Lindenwald R., Mohr L., Günther E., Legler M. (2021). Monitoring of the Infectious Agent Chlamydia psittaci in Common Swifts (*Apus apus*) in the Area of Hannover, Lower Saxony, Germany. Berl. Münch. Tierärztl. Wochenschr..

[32] Gavier-Widén D., Duff J.P., Meredith A. (2012). Infectious Diseases of Wild Mammals and Birds in Europe.

[33] Haupt C. (2001). Mauersegler in Menschenhand: Erste Hilfe—Aufzucht und Pflege—Tierärztliche Versorgung.

[34] Lehmbecker A., Rittinghausen S., Rohn K., Baumgärtner W., Schaudien D. (2014). Nanoparticles and pop-off technique for electron microscopy: A known technique for a new purpose. Toxicol. Pathol..

[35] Pérez-Tris J.A., Williams R.A.J., Abel-Fernández E., Barreiro J., Conesa J.J., Figuerola J., Martinez-Martínez M., Ramírez A., Benitez L. (2011). A Multiplex PCR for Detection of Poxvirus and Papillomavirus in Cutaneous Warts from Live Birds and Museum Skins. Avian Dis..

[36] Husnjak K., Grce M., Magdić L., Pavelić K. (2000). Comparison of five different polymerase chain reaction methods for detection of human papillomavirus in cervical cell specimens. J. Virol. Methods.

[37] van Devanter D.R., Warrener P., Bennett L., Schultz E.R., Coulter S., Garber R.L., Rose T.M. (1996). Detection and Analysis of Diverse Herpesviral Species by Consensus Primer PCR. J. Clin. Microbiol..

[38] Godoy L.A., Dalbeck L.S., Tell L.A., Woods L.W., Colwell R.R., Robinson B., Wethington S.M., Moresco A., Woolcock P.R., Ernest H.B. (2013). Characterization of avian poxvirus in Anna’s Hummingbird (*Calypte anna*) in California, USA. J. Wildl. Dis..

[39] Galvin A.N., Pandit P.S., English S.G., Quock R.C., Bandivadekar R.R., Colwell R.R., Robinson B.W., Ernest H.B., Brown M.H., Sehgal R.N.M. (2022). Evaluation of minimally invasive sampling methods for detecting Avipoxvirus: Hummingbirds as a case example. Front. Vet. Sci..

[40] Lachish S., Bonsall M.B., Lawson B., Cunningham A.A., Sheldon B.C. (2012). Individual and population-level impacts of an emerging poxvirus disease in a wild population of great tits. PLoS ONE.

[41] Attig F., von Dörnberg K., Meyer H., Zoeller G., Baumgärtner W., Wohlsein P. (2020). Shearwater poxvirus infection in a flamingo in Germany; Sturmtaucherpockenvirusinfektion bei einem Flamingo in Deutschland. Berl. Münch. Tierärztl. Wochenschr..

[42] Forrester D.J. (1991). The ecology and epizootiology of avian pox and malaria in wild turkeys. Bull. Soc. Vector Ecol..

[43] Aruch S., Atkinson C.T., Savage A.F., LaPointe D.A. (2007). Prevalence and distribution of pox-like lesions, avian malaria, and mosquito vectors in Kīpahulu valley, Haleakalā National Park, Hawaii, USA. J. Wildl. Dis..

[44] Hattendorf C. (2025). Circulation and Spread of Mosquito-Borne Pathogens in Germany and Europe. Doctoral Dissertation.

[45] Wawman D., Jones B.P., Fiddaman S.R., Turner J.E., Johnson N., Smith A.L. (2025). A first report of the detection of *Avipoxvirus* genomic sequences in louse flies (Diptera: Hippoboscidae). Parasitology.

[46] Buenestado F., Gortázar C., Millán J., Höfle U., Villafuerte R. (2004). Descriptive study of an avian pox outbreak in wild red-legged partridges (*Alectoris rufa*) in Spain. Epidemiol. Infect..

[47] Crawford J.A. (1986). Differential prevalence of avian pox in adult and immature California quail. J. Wildl. Dis..

